# Assessment of Neurobehavioral Performance and Markers of Synaptic Vesicle Trafficking in an Alpha-Synuclein Knockout Mouse Model after Controlled Cortical Impact Injury

**DOI:** 10.1177/2689288X251379063

**Published:** 2025-09-16

**Authors:** Eleni H. Moschonas, Zachary Rohde, Madison Parry, Japna Bhatia, Scott Fellman, Elyse C. Pullen, Ananya R. Shah, Jeremy Henchir, Youming Li, C. Edward Dixon, Shaun W. Carlson

**Affiliations:** ^1^ Department of Neurological Surgery, University of Pittsburgh, Pittsburgh, Pennsylvania, USA.; ^2^ Veterans Affairs Pittsburgh HealthCare System, Pittsburgh, Pennsylvania, USA.

**Keywords:** behavioral assessments, controlled cortical impact, neurotransmitters, synapse, traumatic brain injury

## Abstract

Traumatic brain injury (TBI) is associated with significant deficits across cognitive, emotional, and somatic functions, contributing to reduced quality of life for TBI survivors. Synaptic vesicle cycling, crucial for neurotransmitter release, involves a tightly regulated process of exocytosis and endocytosis, of which monomeric alpha-synuclein (mAS) is implicated in both, and therefore, may underpin neurotransmission impairments post-TBI. Our team previously demonstrated that controlled cortical impact (CCI) reduces hippocampal and cortical mAS in the weeks postinjury. We hypothesized that genetic knockout (KO) of mAS expression may exacerbate TBI-induced deficits in neurobehavioral performance and synaptic health. To elucidate the role of AS in neurobehavioral recovery and histopathological alterations post-TBI, we employed a genetic AS-KO mouse model (B6;129X1-Snca<tm1Ros1>/J). Markers of exocytosis and endocytosis, cysteine-string protein alpha (CSPα) and clathrin light chain (CLC), respectively, and the astrocytic marker glial fibrillary acidic protein (GFAP) were assessed. Male AS-KO and wild-type (WT) littermate controls received 1.8 mm CCI or Sham control surgery (Sham-WT, CCI-WT, CCI-KO, *n* = 10/group). Beam balance testing (1–5 days) did not reveal differences in balance latency between groups. Spatial learning (Morris water maze, 9–13 days) was significantly impaired in both CCI groups relative to Sham-WT controls, whereas the CCI-KO mice performed comparably to Sham-WT mice in spatial memory testing. Following CCI, GFAP expression was significantly elevated in the ipsilateral hemisphere, independent of genotype, compared to Sham controls. CCI also resulted in a reduction of CLC expression in the ipsilateral hemisphere, while CSPα expression remained unchanged in both hemispheres across all groups.

## Introduction 

Traumatic brain injury (TBI) is a major public health concern, frequently resulting in chronic motor, cognitive, and affective impairments. Beyond the immediate mechanical insult, TBI initiates a complex secondary cascade characterized by excitotoxicity, oxidative stress, neuroinflammation, and progressive neurodegeneration. A key pathological feature emerging across these stages is synaptic dysfunction, which compromises neurotransmission and neural circuit integrity in regions critical for motor coordination and cognitive processes. Notably, synaptic disruptions following TBI have been implicated in increasing the risk of later-life neurodegenerative diseases, including Parkinson’s disease (PD).^1–5^

Among the cellular processes disrupted after TBI, synaptic vesicle cycling is essential for maintaining neurotransmission and neural network integrity.^6–12^ This tightly regulated process involves synaptic vesicle exocytosis of neurotransmitters into the synaptic cleft and endocytic recycling to replenish vesicle pools for sustained synaptic activity.^6,7,13^ Experimental and transcriptomic studies indicate that TBI disrupts synaptic vesicle cycling through both structural and molecular mechanisms.^8–12^ Reductions in key synaptic vesicle proteins, including synapsin-I, synaptic vesicle glycoproteins SV2A and SV2B, and SNARE complex components have been observed in injured brain regions such as the hippocampus and cortex.^8–10,12^ These alterations are associated with impaired SNARE complex formation, depletion of the readily releasable vesicle pool, and compromised neurotransmitter release.^8^ Furthermore, transcriptomic analyses reveal downregulation of genes involved in vesicle trafficking and glutamate signaling following mild and repetitive TBI.^11^ Together, these findings highlight synaptic vesicle cycling as a key pathological target after TBI, with its disruption likely contributing to the long-term observed functional and cognitive impairments.

Alpha-synuclein (α-synuclein), a presynaptic protein enriched at neuronal terminals, has been implicated in both phases of vesicle cycling.^14–17^ In its monomeric form (mAS), α-synuclein facilitates synaptic vesicle docking and neurotransmitter release by promoting SNARE complex assembly.^18^ This function is supported by its interaction with cysteine-string protein alpha (CSPα), a synaptic vesicle-associated co-chaperone critical for SNARE complex stabilization and exocytotic efficiency.^19–20^ In addition to its role in exocytosis, α-synuclein also supports endocytosis through interactions with phospholipid membranes and components of the clathrin-mediated pathway, including clathrin light chain (CLC).^13,21,22^ While α-synuclein has been extensively studied in neurodegenerative disease, particularly PD, its role in synaptic maintenance under physiological and pathological conditions, such as after TBI, remains understudied. Notably, previous work from our laboratory demonstrated a time-dependent reduction in mAS levels within the ipsilateral hippocampus, striatum, and cortex in the weeks following controlled cortical impact (CCI), a well-established rodent model of TBI.^23^

Given the importance of mAS in maintaining SNARE-dependent neurotransmission, the loss of α-synuclein could impair vesicle dynamics and exacerbate synaptic dysfunction following injury. However, the extent to which α-synuclein deletion influences synaptic plasticity and glial responses in the injured brain remains unclear. In this study, we employed a full genetic knockout (KO) model of α-synuclein (B6;129X1-Snca^tm1Rosl^/J) to examine how the absence of mAS affects behavioral and molecular outcomes after CCI. We hypothesized that α-synuclein KO mice would exhibit greater impairments in motor and cognitive performance, along with altered expression of synaptic vesicle cycling proteins and astrocytic reactivity.

## Methods

### Animal welfare

All experimental procedures were approved by the Institutional Animal Care and Use Committee at the University of Pittsburgh and conducted in accordance with the National Institutes of Health Guide for the Care and Use of Laboratory Animals. Adult (8–12 weeks old) male genetic α-synuclein-targeted knockout (KO; B6;129X1-Snca<tm1Ros1>/J, RRID:IMSR_JAX:003692; *n* = 10)^24^ and WT (B6129SF2/J, RRID:IMSR_JAX:101045; *n* = 20) littermate mice (Jackson Laboratories) were group-housed in a temperature-controlled environment (21 ± 1°C) with a 12-h light/dark cycle (lights on: 07:00 AM–07:00 PM). Breeding, genotyping and identification of the KO mouse and wild-type control strains were completed by Jackson Laboratories. Once received from Jackson Laboratories, animals had *ad libitum* access to food and water in the University of Pittsburgh vivarium.

## Controlled Cortical Impact

Mice were randomly assigned to one of two experimental groups: WT Sham (*n* = 10) or WT CCI (*n* = 10), while all the KO mice were assigned to the CCI group (*n* = 10). Anesthesia was induced and maintained via nose cone with 4% and 2% isoflurane, respectively, in a 2:1 N_2_O/O_2_ mixture. Mice were positioned in a stereotaxic frame in the prone position, and core body temperature was monitored and maintained at 37 ± 0.5°C using a rectal thermistor and heating pad. Under aseptic conditions, a midline scalp incision was made, and soft tissues were retracted to expose the skull. A 5-mm craniectomy was performed over the right parietal cortex (AP: −3.0 mm, ML: +3.0 mm from lambda) using a dental drill, exposing the dura mater. A pneumatic-controlled cortical impactor (Pittsburgh Precision Instruments Inc., Pittsburgh, PA) with a flat 3 mm tip was positioned over the craniectomy site, ensuring contact with the exposed dura. The impactor was then advanced to a depth of 1.8 mm at a velocity of 6 m/s with a dwell time of 150 ms. Following the impact, anesthesia was discontinued, the incision was sutured, and mice were assessed for righting time. Sham control mice underwent identical surgical procedures but did not receive an impact. Once the effects of anesthesia abated and mice regained ambulation, they were returned to their home cages. Postoperatively, all mice were provided with standard chow mush (milled chow mixed with water) *ad libitum* for 5 days following surgery. Pre- and postsurgical weights were recorded on the motor and cognitive assessment days.

## Beam Balance

The beam balance task was used to assess motor function following Sham or CCI surgery. One day prior to surgery, all mice underwent training on the beam balance task and preassessed (i.e., “Pre”) immediately prior to surgery. Postoperative beam balance performance was assessed on days 1–5 following surgery. Mice were positioned on a suspended 0.25-inch cylindrical beam, elevated 30 inches above a padded surface. Latency to fall (sec) was recorded across three trials per day, with a 30 sec intertrial interval. The maximum trial duration was 60 sec.

### Morris water maze

The Morris water maze (MWM) task was used to assess spatial learning and memory on postoperative days 9–13 and 14, respectively, as previously described.^25^ The apparatus consisted of a circular pool (100 cm in diameter, 60 cm in height) filled with 26±°C water to a depth of 30 cm. A transparent circular platform (4 cm in diameter, 29 cm in height) was submerged 1 cm below the water surface and positioned 15 cm from the wall in the southwest quadrant. The testing room contained salient extra-maze cues to facilitate spatial navigation.

Spatial learning was evaluated across 5 consecutive days (postoperative days 9–13), during which mice underwent four daily trials (4-min intertrial interval). Each mouse was allowed a maximum of 120 sec per trial to locate the hidden platform. Between trials, mice were placed in a 37°C incubator for a 4-min rest period. On postoperative day 14, a single probe trial was conducted to assess spatial memory. The platform was removed, and mice were allowed to swim freely for 60 sec. On postoperative day 14, a 60 sec visible platform test was performed to evaluate visual function.

MWM data were recorded using ANY-maze video tracking software (Stoelting, Inc., Wood Dale, IL, USA). Dependent measures included: (1) latency (s) to locate the hidden platform during training (i.e., spatial acquisition), (2) percentage of time spent in the target quadrant during the probe trial (i.e., spatial memory), and (3) latency (s) to locate the visible platform (i.e., visual function).

### Histological and immunohistochemistry

Following the completion of MWM (i.e., postinjury day 14), mice received an overdose of Fatalplus (intraperitoneally, 0.1 mL/kg sodium pentobarbital) and were transcardially perfused with saline, followed by 10% neutral-buffered formalin (Fisher Scientific, Waltham, MA). The brains were extracted, cryoprotected in 15%, then 30% sucrose in phosphate-buffered saline, then frozen, embedded in Tissue-Plus O.C.T. compound (Fisher Scientific), and stored at −80°C until sectioning. For histological analysis, brain sections spanning 0.0 to −4.0 mm relative to bregma were collected at 35 μm intervals and mounted onto gelatin-coated slides. Sections were cleared in xylene, rehydrated through a graded ethanol series, rinsed in distilled water, and stained with cresyl violet. Following staining, slides were dehydrated, cleared in xylene, and cover-slipped using Permount mounting medium.

For immunohistochemical analysis, sections from the same mice used for behavioral and histopathological assessments were selected, spanning −1.9 to −2.3 mm relative to bregma, encompassing the lesion site. Staining was performed on free-floating sections in 6-well plates, with one section per mouse designated for each target protein. Sections were rinsed in 0.1 M Tris-buffered saline (TBS) containing 0.1% Triton X-100 (TBS-T) and blocked with 10% normal goat serum in TBS-T for 1 h at room temperature (RT).

Sections were incubated overnight at 4°C with primary antibodies against glial fibrillary acidic protein (GFAP; Rabbit, 1:1000, Millipore, Cat# AB5804, RRID:AB_2109645), clathrin light chain isoform A (CLC; Rabbit, 1:1000 Invitrogen, Cat# PA34670, RRID:AB_2552022), and cysteine-string protein alpha (CSPα; Rabbit, 1:1000, Synaptic Systems, Cat# 154003, RRID:AB_887710). The following day, sections were rinsed three times for 5 min each in TBS-T, then incubated with a biotinylated secondary antibody for 1 h at RT. After an additional three washes in TBS-T (5 min each), sections were incubated with horseradish peroxidase (HRP) conjugate. Staining was visualized using diaminobenzidine (DAB, Vector Laboratories, Burlingame, CA), with reaction development time standardized across all sections for each protein stain. Finally, sections were washed (3 × 5 min in TBS-T), mounted on gelatin-coated slides, and cover-slipped using Permount mounting medium. Sham and CCI tissues were processed simultaneously to ensure consistency.

Brain hemispheric tissue loss was analyzed using the program morphometric image analysis (Imaging Research, St. Catherines, Ontario, Canada), as previously described.^8^ Ipsilateral tissue loss is expressed as a percentage of contralateral and calculated as (contralateral-ipsilateral)/contralateral × 100. To quantify differences in pixel intensity in immunohistochemical staining between Sham and CCI-injured brains, photomicrographs of brain sections were acquired at 10× magnification using a Nikon C2 90i microscope. Images of GFAP, CLC, and CSPα staining were imported into ImageJ and inverted to obtain mean pixel intensity values. The polygon tool was used to delineate the contralateral and ipsilateral hippocampus, and the mean pixel intensity for each region was measured. Background normalization was performed by subtracting the mean pixel intensity of the slide from the mean pixel intensity of each hemispheric region. For both the ipsilateral and contralateral dorsal hippocampus, mean pixel intensity values were averaged and normalized as a percentage of the corresponding Sham intensity.

### Statistics

All data were collected by experimenters blinded to injury and genotype conditions. Data are presented as mean ± standard error of the mean (±SEM). Statistical analyses were conducted using GraphPad Prism (version 9.1, GraphPad Software, La Jolla, CA). Postsurgical righting times, spatial memory (i.e., probe), hemispheric tissue loss, and immunohistochemical staining were analyzed using one-way ANOVA. Statistical comparisons of body weight, beam balance performance, and spatial learning were assessed using a repeated measures one-way ANOVA (rmANOVA) comparing time and injury status. When overall ANOVAs revealed a significant effect, Tukey’s *post hoc* test was applied to determine group differences. A *p-value* of <0.05 was considered statistically significant for all measures.

A total of 30 mice were initially assigned to experiment groups (WT Sham = 10; WT CCI = 10, and KO CCI = 10). One mouse in the WT Sham group died during postsurgical recovery and was excluded from all analyses. One mouse from the WT Sham group was excluded from all behavioral assessments (i.e., beam balance and MWM) due to the inability to locate the visible platform, suggesting a noncognitive impairment (e.g., vision or motivation deficits). As a result, the final group sizes for the behavioral data are WT Sham = 8; WT CCI = 10, and KO CCI = 10. For histological assessments, one mouse from the WT CCI group was excluded due to poor tissue quality resulting from storage. Therefore, the final groups for hemispheric tissue loss and immunohistochemistry are WT Sham = 9; WT CCI = 9, and KO CCI = 10. Final group sizes are reported in the figure legends.

## Results

### Physiological: Acute neurological assessment and weights

**FIG. 1.**
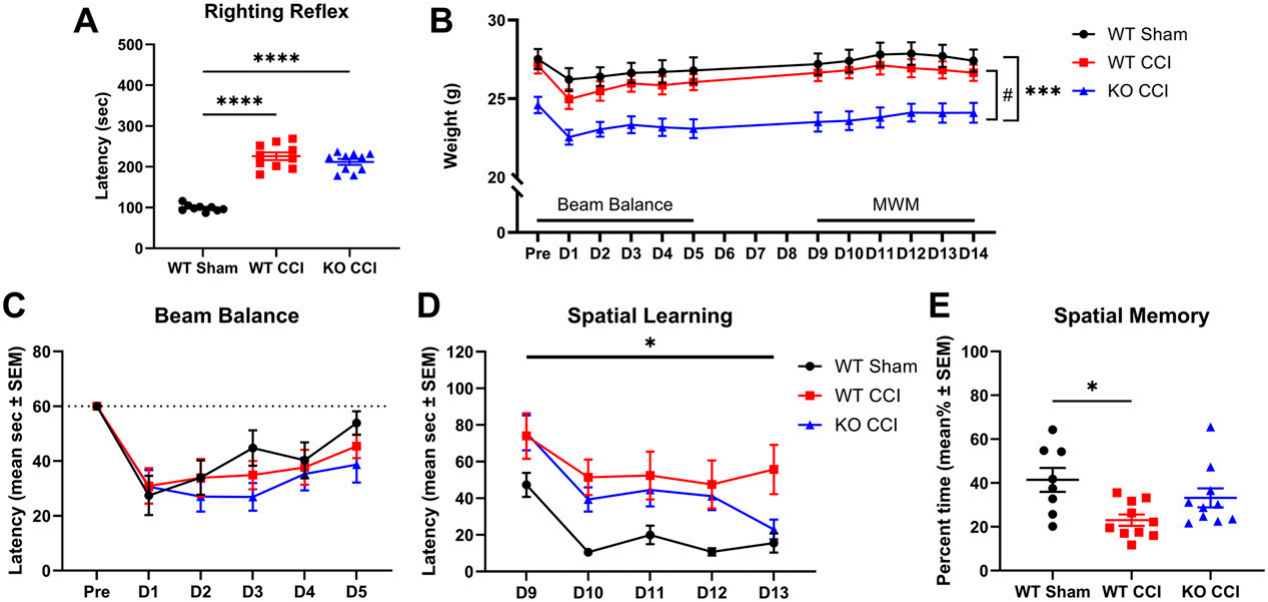
**(A)** Postsurgical latency to right was significantly increased in both WT CCI and KO CCI groups compared to WT Sham controls (one-way ANOVA with Tukey’s *post hoc* test, ****p < 0.0001), with no significant difference between the injured groups (WT CCI vs. KO CCI, *p* > 0.05). **(B)** Body weights recorded presurgically and across the testing period (beam balance: D1–D5; MWM: D9–D14) revealed genotype-specific differences, with KO CCI mice weighing less than both WT CCI and WT Sham mice throughout testing (****p < 0.0001). Additionally, CCI induced a reduction in weight relative to Sham controls (*p* < 0.0001). The groups for righting reflex and weights are WT Sham (*n* = 9), WT CCI (*n* = 10), KO CCI (*n* = 10). **(C)** Beam balance performance across 5 consecutive days did not show a significant difference (rmANOVA, *p* > 0.05) between groups. The dotted line indicates the maximum latency allowed (i.e., 60 sec). **(D)** Spatial learning assessed from postoperative days 9–13 using the MWM revealed that CCI impaired spatial learning regardless of genotype (rmANOVA with Tukey’s *post hoc* test; WT CCI vs. KO CCI, *p* > 0.05; WT Sham vs. injured groups, *p < 0.05). **(E)** Spatial memory performance, assessed on postoperative day 14 by percent time spent in the target quadrant, showed that WT CCI mice exhibited significant impairments relative to WT Sham controls (one-way ANOVA, *p < 0.05). KO CCI mice did not differ from WT Sham controls (*p* > 0.05). Behavioral groups are WT Sham (*n* = 8), WT CCI (*n* = 10), KO CCI (*n* = 10). ANOVA, analysis of variance; CCI, controlled cortical impact; KO, knockout; rmANOVA, repeated measures one-way ANOVA; WT, wild-type.

Acute assessment of righting reflex (Fig. 1A) revealed a significant group effect [F_(2,26)_ = 88.5; *p* < 0.05]. Tukey’s *post hoc* analysis revealed a significant increase in latency to right in both CCI groups compared to the Sham control (WT CCI and KO CCI vs. Sham, *p* < 0.05), albeit no significant differences were revealed between the CCI groups (WT CCI vs. KO CCI, *p* > 0.05), indicating that both injury groups experienced similar levels of anesthesia and injury severity.

Prior to surgery, the mean body weights (±SEM) were 27.52 ± 0.64 g for WT Sham, 27.11 ± 0.50 g for WT CCI, and 24.60 ± 0.51 g for KO CCI (Fig. 1B). A rmANOVA revealed significant effects of group [F_(1.755, 19.30)_ = 1118, *p* < 0.0001] and Day [F_(11, 22)_ = 28.34, *p* < 0.0001] effects. Tukey’s *post hoc* analysis showed no significant difference between the WT Sham and WT CCI groups (WT Sham vs. WT CCI, *p* > 0.05); however, the KO CCI group had significantly lower weights compared to both WT Sham and WT CCI groups (KO CCI vs. WT Sham and WT CCI; *p* < 0.0001).

### Neurobehavioral recovery

#### Motor

Assessment of vestibulomotor function using the beam balance task demonstrated that all mice were able to maintain balance on the beam for the full 60 s duration prior to CCI, indicating intact motor coordination across groups at baseline (Fig. 1C). Following surgery, a rmANOVA revealed no significant differences between groups [F_(1.082, 5.410_) = 4.07, *p* = 0.094].

#### Morris Water Maze

Repeated measures analysis of escape latency during spatial acquisition training revealed significant main effects of group [F_(1.237, 4.947_) = 33.69, *p* < 0.005] and Day [F_(4, 8)_ = 12.68, *p* < 0.005] (Fig. 1D). Tukey’s *post hoc* analysis demonstrated that both the WT CCI and KO CCI groups exhibited significantly longer escape latencies compared to WT Sham controls (WT Sham vs. WT CCI, *p* < 0.0005; WT Sham vs. KO CCI, *p* < 0.05), suggesting TBI-induced impairments in spatial learning. There were no statistically significant differences between the injured groups (WT CCI vs. KO CCI, *p* > 0.05).

Assessment of spatial memory performance, as measured by time spent in the target quadrant during the probe trial, also revealed a significant effect of group [F_(2, 25)_ = 4.82, *p* < 0.05] differences (Fig. 1E). Tukey’s *post hoc* analysis demonstrated that the WT CCI group spent significantly less time in the target quadrant compared to WT Sham controls (WT CCI vs. WT Sham, *p* < 0.05), indicating impaired memory retention. The KO CCI group, however, did not differ significantly from either WT Sham (KO CCI vs. WT Sham, *p* > 0.05) or WT CCI mice (KO CCI vs. WT CCI, *p* > 0.05), suggesting that the α-synuclein deletion may mitigate TBI-induced memory deficits. There were no significant differences in latency to locate the visible platform across groups [F _(2, 25)_ = 3.13; *p* > 0.05], indicating intact vision function.

### Histopathological alterations

#### Ipsilateral Hemispheric Tissue Loss

**FIG. 2.**
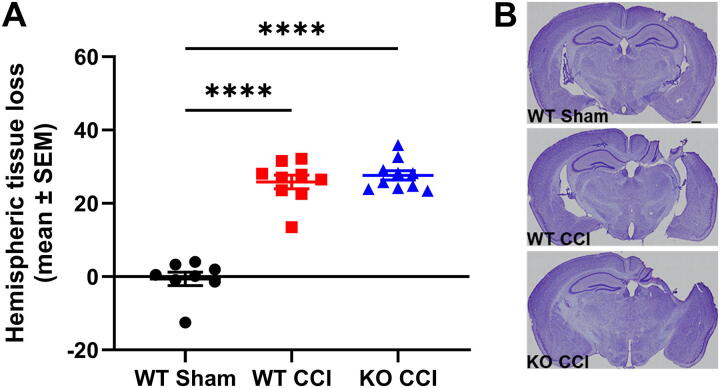
**(A)** Quantification of hemispheric tissue loss revealed a significant group effect, with both the WT CCI and KO CCI groups exhibiting substantial ipsilateral cortical atrophy 14 days postinjury compared to WT Sham controls (one-way ANOVA, **p < 0.0001). **(B)** Representative cresyl violet-stained coronal brain sections (4x magnification) from each group illustrate the extent of ipsilateral tissue loss following CCI. Groups: WT Sham (*n* = 9), WT CCI (*n* = 9), and KO CCI (*n* = 10). Scale bar = 0.50 mm. ANOVA, analysis of variance; CCI, controlled cortical impact; KO, knockout; WT, wild-type.

Quantitative analysis of ipsilateral hemispheric tissue loss using one-way ANOVA revealed a robust group effect within the ipsilateral hemisphere [F_(2, 24)_ = 86.41*, p* < 0.001] (Fig. 2). Tukey’s *post hoc* confirmed significantly greater tissue loss in both WT CCI and KO CCI groups relative to Sham controls (WT CCI vs. WT Sham, *p* < 0.0001; KO CCI vs. WT Sham, *p* < 0.001), with no significant differences between the injured groups (WT CCI vs. KO CCI, *p* > 0.05).

#### GFAP Immunoreactivity

**FIG. 3.**
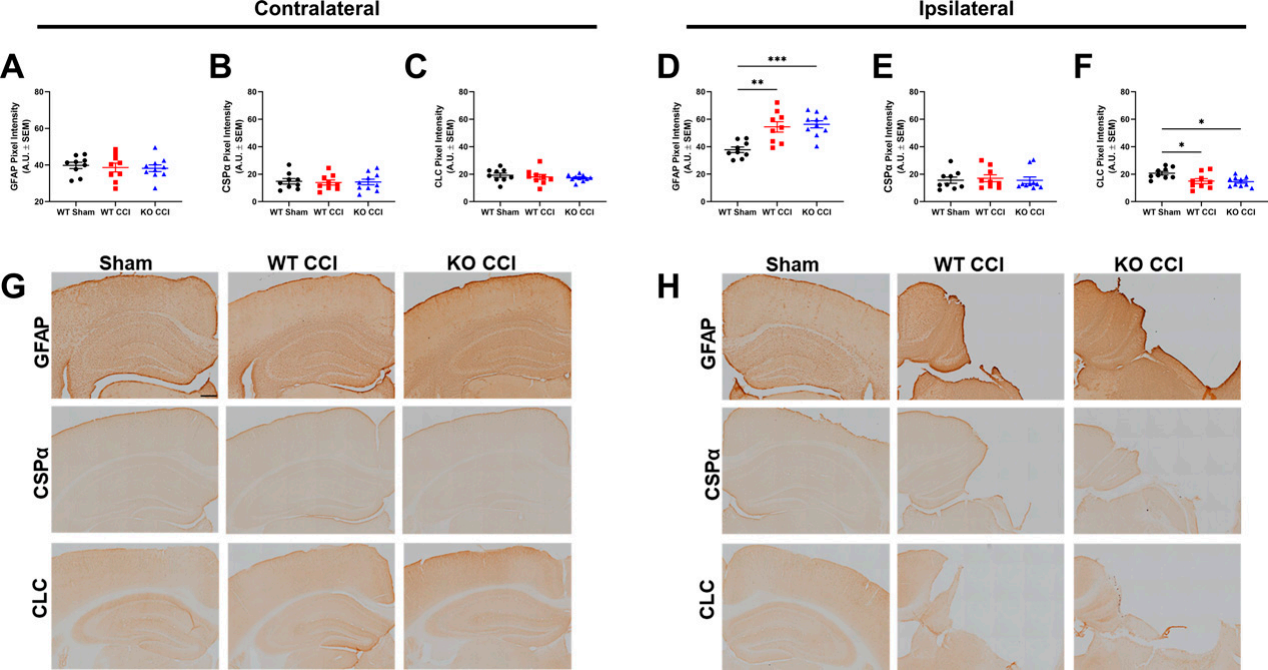
Mean pixel intensity for GFAP, CSPα, and CLC in the contralateral **(A–C)** and ipsilateral **(D–F)** dorsal hippocampus. No significant group differences were detected in GFAP **(A)**, CSPα **(B)**, or CLC **(C)** intensity within the contralateral dorsal hippocampus across groups (one-way ANOVA, all *p* > 0.05). **(D)** In the ipsilateral dorsal hippocampus, GFAP intensity was significantly increased in both WT CCI and KO CCI groups compared to WT Sham (one-way ANOVA, **p < 0.001, ***p < 0.0001). **(E)** CSPα intensity in the ipsilateral dorsal hippocampus did not differ significantly between groups (one-way ANOVA, *p > 0.05). **(F)** CLC intensity was significantly reduced in the ipsilateral dorsal hippocampus in both injured groups compared to WT Sham, independent of genotype (one-way ANOVA, *p < 0.05). **(G)** Representative 10x photomicrographs of GFAP, CSPα, and CLC staining in the contralateral dorsal hippocampus from WT Sham, WT CCI, and KO CCI groups. **(H)** Representative 10x photomicrographs of GFAP, CSPα, and CLC staining in the ipsilateral dorsal hippocampus from WT Sham, WT CCI, and KO CCI groups. Groups: WT Sham (*n* = 9), WT CCI (*n* = 9), KO CCI (*n* = 10). Scale bar = 50 pixels. ANOVA, analysis of variance; CCI, controlled cortical impact; CLC, clathrin light chain; CSPα, cysteine-string protein alpha; GFA, glial fibrillary protein; KO, knockout; WT, wild-type.

GFAP immunoreactivity exhibited a hemispheric-dependent response to injury (Fig. 3A, D, G, H). Analysis of the contralateral dorsal hippocampus revealed no significant group differences [F_(2, 25)_ = 0.17, *p* > 0.05] (Fig. 3A). In contrast, the ipsilateral dorsal hippocampus, one-way ANOVA revealed a significant main effect of group [F_(2, 25)_ = 12.58, *p* < 0.001] (Fig. 3D). Tukey’s *post hoc* analysis demonstrated significantly increased GFAP signal intensity in both the WT CCI (WT CCI vs. WT Sham, *p* < 0.01) and KO CCI (KO CCI vs. WT Sham, *p* < 0.001) groups compared to Sham controls, with no significant difference between the injured groups (WT CCI vs. KO CCI, *p* > 0.05).

#### CSPα Immunoreactivity

Examination of CSPα immunoreactivity by one-way ANOVA revealed no significant differences in CSPα signal intensity within either the ipsilateral hemisphere [F_(2, 25)_ = 0.12, *p* > 0.05] or contralateral [F_(2, 25)_ = 0.05, *p* > 0.05] hemisphere (Fig. 3B, E, G, H).

#### CLC Immunoreactivity

Assessment of CLC immunoreactivity revealed no significant group differences in the contralateral hemisphere [F_(2, 25)_ = 0.48, *p* > 0.05] CLC immunoreactivity (Fig. 3C, G). One-way ANOVA revealed a significant group effect on CLC immunoreactivity within the ipsilateral hemisphere [F _(2, 25)_ = 5.58, *p* < 0.005] (Fig. 3F, H). Tukey’s *post hoc* analysis showed significantly reduced CLC signal intensity in both WT CCI (WT CCI vs. WT Sham, *p* < 0.05) and KO CCI (KO CCI vs. WT Sham, *p* < 0.01) groups relative to Sham controls, with no significant difference between the injured groups (WT CCI vs. KO CCI, *p* > 0.05).

## Discussion

In this study, we investigated the impact of α-synuclein deletion on behavioral and histological outcomes following moderate-to-severe CCI. CCI produced robust cognitive impairments, cortical tissue loss, reactive astrogliosis, and reductions in clathrin-mediated endocytosis pathways. However, genetic deletion of mAS did not exacerbate these injury-induced deficits. Instead, the KO CCI group did not differ from either the WT Sham or the WT CCI groups in hippocampal-dependent spatial memory during the subacute recovery phase, suggesting that loss of mAS may engage compensatory synaptic mechanisms that mitigate TBI-induced cognitive dysfunction without influencing gross pathological outcomes such as tissue loss, astrocytic activation, or synaptic vesicle cycling disruption.

Motor performance, as assessed by beam balance testing, was comparable between all groups. While there was a trend toward group differences (*p* = 0.09) that included both the WT CCI and KO CCI groups, this did not reach significance. This suggests that neither injury nor mAS deletion was influenced by gross motor recovery after injury. Although this finding is unexpected following CCI, alternative motor tasks that are more complex, such as beam walk, which requires traversing across a narrow beam, may reveal fine vestibular motor deficits that are not detected by the beam balance task. Our laboratory has previously demonstrated that following repeated lateral fluid percussion injury in adult male rats no compounded motor impairment as assessed by beam balance, however, vestibular motor function in the beam walking task revealed significant differences in performance as a function of repeated injury (i.e., dual and quad fluid percussion injury) compared to Sham controls, suggesting task-specific and species-specific sensitivity to injury severity.^12^ One mouse from the WT Sham group was excluded from all behavioral analyses, including beam balance testing, due to failure to locate the visible platform during MWM testing. Using our established *a priori* statistical guidelines, failure to locate the visible platform was used as an exclusion criterion to account for potential noncognitive impairments. Notably, the lack of early motor deficits in this cohort contrasts with our previous findings, which demonstrate that disruptions in synaptic vesicle docking and SNARE complex formation contribute to early functional impairments following experimental TBI in a rat model of CCI.^8^ Although restoration of SNARE complex proteins improves cognitive but not early motor outcomes.^9,10,12^ A study from Konan et al. showed that similar deficits in presynaptic vesicle properties are also impaired in the month following low-intensity blast TBI, suggesting these deficits may extend across the injury spectrum.^26^ These findings suggest that early motor deficits after TBI likely involve broader injury mechanisms beyond synaptic vesicle cycling alone.

In contrast, spatial memory performance revealed a genotype-dependent effect. While both WT CCI and KO CCI groups exhibited impaired spatial learning during acquisition trials, memory retention during probe testing did not significantly differ between the KO CCI and either the WT Sham or CCI groups. Fronczak and colleagues demonstrated that CCI-induced reductions in presynaptic proteins such as SV2A and SV2B coincide with motor and cognitive impairments, emphasizing the critical role of synaptic vesicle maintenance in functional recovery after TBI.^12^ Although we similarly observed TBI-induced impairments, the intact memory retention in the KO CCI group suggests that compensatory synaptic adaptations can contribute to the mitigation of injury-induced deficits, potentially via reduced vesicle disruption. Mechanistically, mAS facilitates synaptic vesicle cycling by promoting SNARE complex assembly and maintaining vesicle pool dynamics.^16,27^ In the absence of mAS, alternative presynaptic mechanisms, including enhanced CSPα function or compensatory activity of β- and γ-synuclein isoforms, may support neurotransmitter release after injury.^19,20^ Alternatively, deletion of α-synuclein may mitigate synaptic dysfunction by removing a protein that, under pathological conditions, has been implicated in impairing synaptic vesicle dynamics, thereby preserving cognitive function after TBI.^15,17,28^

Histopathological analyses revealed that both WT CCI and KO CCI groups exhibited robust cortical tissue loss and reactive astrogliosis within the ipsilateral dorsal hippocampus, hallmark features of moderate-to-severe CCI. GFAP expression was comparably elevated between genotypes, indicating that mAS deletion did not influence the magnitude of astrocytic reactivity. Although astrocyte activation is primarily driven by biomechanical injury and inflammatory processes, emerging evidence suggests that neuronal dysfunction, particularly disruptions in synaptic vesicle cycling, may further amplify glial responses.^29,30^ In this context, the observed reductions in CLC expression across both WT CCI and KO CCI groups suggest impairments in clathrin-mediated endocytosis, potentially disrupting vesicle recycling and neurotransmitter clearance and thereby contributing to astrocytic activation. Although we did not directly assess extracellular neurotransmitter dynamics, impaired endocytosis may represent an additional mechanism of neuronal vulnerability following TBI. Nevertheless, the preservation of CSPα expression across groups suggests that presynaptic chaperone systems are maintained following mAS deletion and injury, potentially supporting residual synaptic function despite broader impairments in vesicle trafficking. No significant differences were detected in contralateral markers, consistent with the focal nature of the cortical insult to the right parietal cortex.

Body weight differences between groups represent a potential confound; the modestly lower weight observed in the KO CCI group relative to WT CCI controls is consistent with prior descriptions of synuclein-targeted models.^31^ However, no genotype-dependent differences in lesion volume and righting times were observed, indicating that weight disparities did not influence injury severity in this model. An apparent limitation of the present study is the absence of a KO Sham control group, precluding full dissociation of baseline genotype effects from injury-induced changes. Future studies incorporating Sham KO controls and longer-term postinjury assessments will be critical to delineate the intrinsic consequences of α-synuclein deletion and to determine whether the cognitive resilience observed here persists into the chronic phase of recovery, as synaptic adaptations may diminish with aging.^32^

In conclusion, these findings demonstrate that while moderate-to-severe TBI induces substantial synaptic, glial, and cognitive impairments, genetic deletion of mAS does not exacerbate these outcomes and is associated with partial preservation of hippocampal-dependent memory. These results suggest that mAS contributes to TBI-induced cognitive vulnerability, potentially through its physiological role in synaptic vesicle trafficking, docking, fusion, and recycling. Therefore, targeting presynaptic mechanisms that stabilize synaptic function may therefore represent a promising strategy for mitigating cognitive dysfunction following TBI.

## Abbreviations Used

α-synuclein: alpha-synuclein
CLC: clathrin light chain
CCI: controlled cortical impact
CSPα: cysteine-string protein alpha
GFAP: glial fibrillary acidic protein
HRP: horseradish peroxidase
KO: knockout
mAS: monomeric alpha synuclein
MWM: Morris water maze
PD: Parkinson’s disease
rmANOVA: repeated measures one-way ANOVA
RT: room temperature
TBI: traumatic brain injury
TBS: tris-buffered saline
WT: wild-type
